# The double ACL sign: An aberrant bucket-handle tear of lateral meniscus

**DOI:** 10.1016/j.amsu.2020.04.006

**Published:** 2020-04-25

**Authors:** Adel A. Al-Ahaidib, Hamza M. Alrabai, Ahmed Alajlan, Yasser Al-shehab, Abdulaziz S. Al-Ahaideb

**Affiliations:** a Department of Orthopaedic Surgery, King Saud University, Riyadh, Saudi Arabia; b Department of Orthopedics, College of Medicine, King Saud University, Riyadh, Saudi Arabia

**Keywords:** Double ACL sign, Bucket-handle tear, Lateral meniscus, Knee, Case report

## Abstract

**Introduction:**

Meniscal injuries are one of the most common musculoskeletal injuries around the knee affecting patients of different genders, ages and activity levels. These injuries could be acute or chronic tears that cause pain and mechanical symptoms based on the injury severity and whether it is displaced and entrapped in an abnormal location within the knee or not. Advances in magnetic resonance imaging (MRI) allowed us to have a better understanding of multiple bucket handle meniscal tear patterns with its specific MRI signs which have been reported in the literature.

**Case presentation:**

This report presents a rare case of a 16-year-old boy with atypical bucket-handle tear of lateral meniscus and MRI showed a bucket-handle tear of lateral meniscus with a fragment entrapped behind and parallel to the anterior cruciate ligament (ACL) appearing as another ACL in sagittal views. Meniscus was repaired arthroscopically.

**Conclusion:**

In our case, the unique and infrequent mechanism led to a bucket-handle tear involving lateral meniscus with a meniscal fragment entrapped in an unusual place intra-articularly behind ACL giving the appearance of a rare MRI sign “double ACL sign”. However, double ACL sign secondary to lateral meniscal tear has been reported only once previously up to the authors’ knowledge.

## Highlights

• Radiologically, meniscal tears have multiple pathognomatic signs.
• Double acl sign was reported twice only, one from medial meniscus and the other from lateral meniscus.
• Double acl sign indicates huge bucket handle tear which displaced into the intercondylar notch.

## 1 Introduction

Meniscal injuries are one of the most common musculoskeletal injuries around the knee affecting patients of different genders, ages and activity levels [1,2]. These injuries could be acute or chronic tears that cause pain and mechanical symptoms based on the injury severity and whether it is displaced and entrapped in an abnormal location within the knee or not [3]. Advances in MRI allowed us to have a better understanding of multiple bucket handle meniscal tear patterns with its specific MRI signs which have been reported in the literature [[4], [5], [6], [7], [8], [9], [10]]. We aim in this case report to present a characteristic MRI sign of bucket handle tear of lateral meniscus with a meniscal fragment being sequestered behind the ACL resulting in the double ACL sign. Double ACL sign secondary to lateral meniscal tear has been reported only once by Bui-Mansfield and DeWitt [11]. This case has been reported in line with the Surgical Case Report Guidelines (SCARE) 2018 criteria [12].

The patient's parents were informed that the clinical data concerning the case would be submitted for publication and they provided informed consent.

## 2 Case report

A 16 year old boy who twisted his left knee while trying to stand up at a brisk pace from a sitting position on floor. Instantaneously, he heard a popping sound associated with pain and swelling. At emergency department in our institute, initial examination was limited because of knee swelling and guarding due pain.

**Fig. 1:**
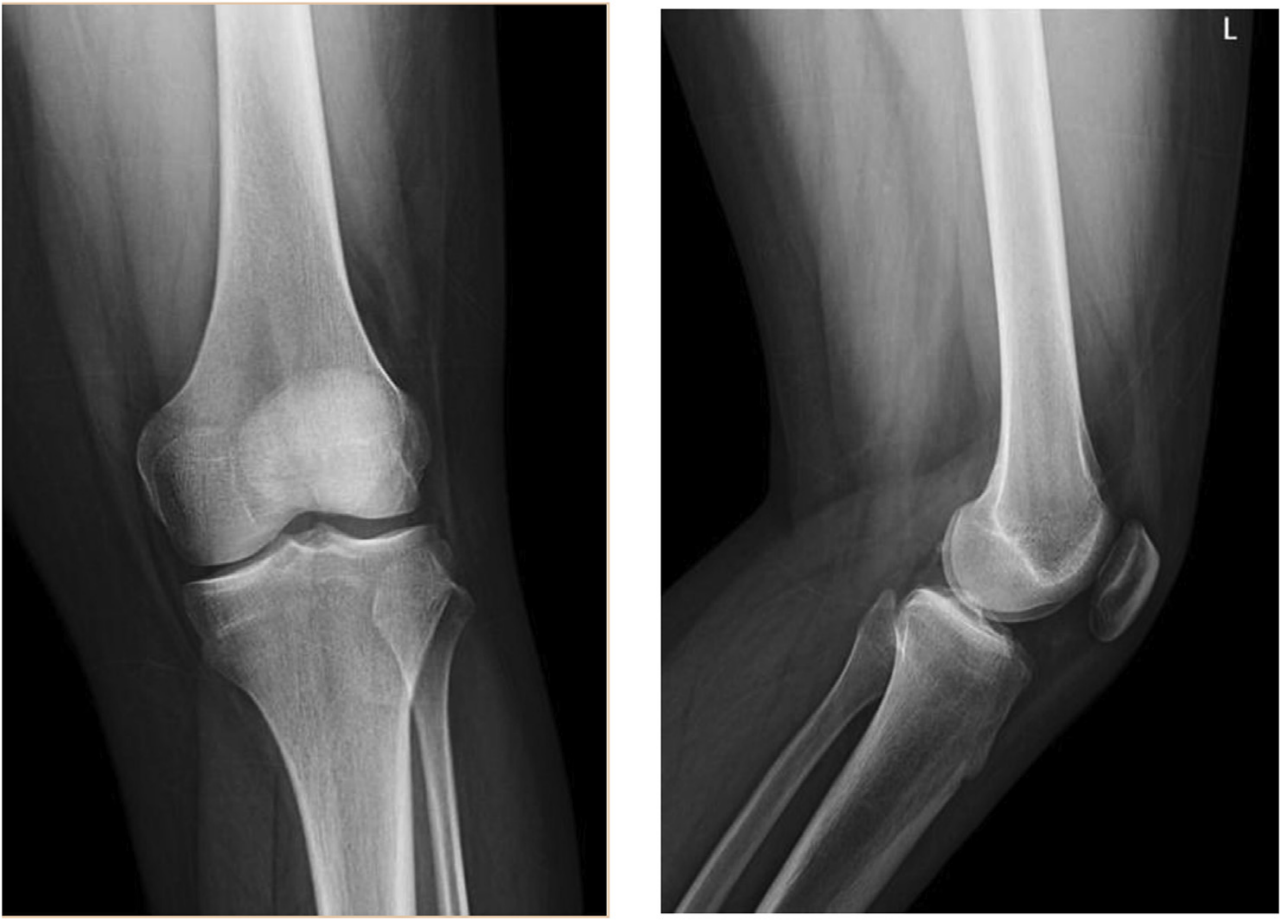
Anteroposterior and lateral x-ray views of left knee were reported as normal.

**Fig. 2:**
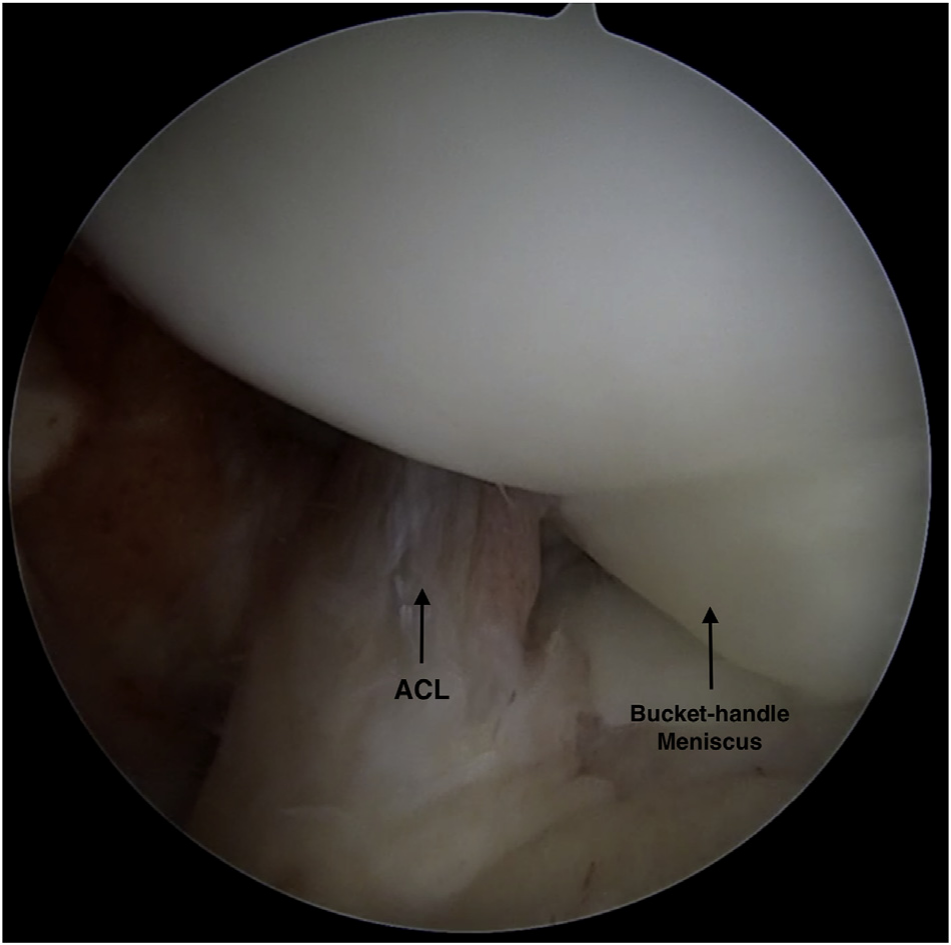
MRI of left knee. **A)** T2 weighted coronal view shows a meniscal fragment within the intercondylar notch. **B)** T2 sagittal view shows vertical tear in the peripheral zone of lateral meniscus. **C)** T2 sagittal view with an arrow points to the meniscal fragment entrapped behind ACL forming the double ACL sign. **D)** T1 weighted coronal view shows a meniscal fragment parallel to ACL within the notch.

**Fig. 3:**
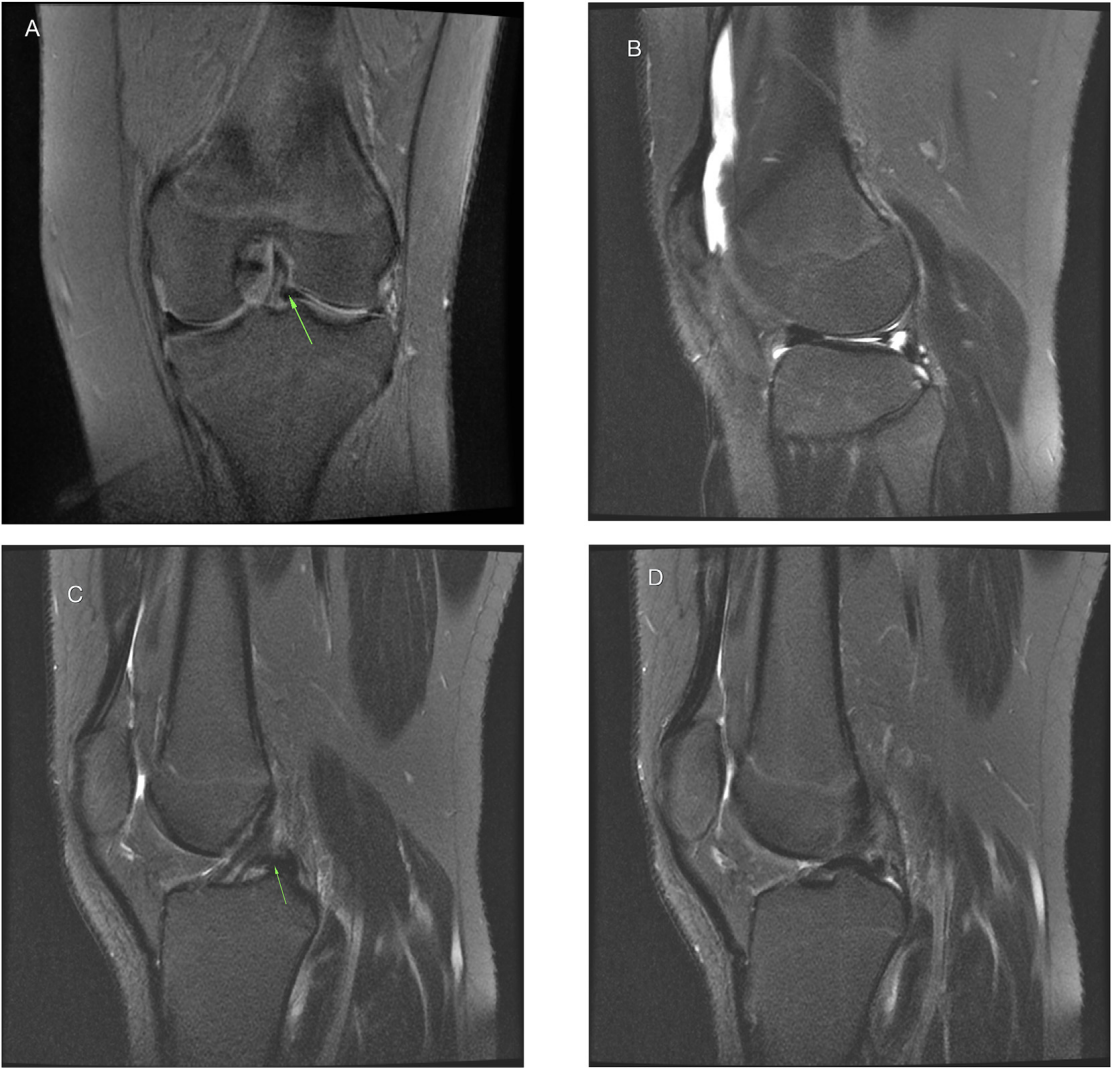
An arthroscopic view of left knee shows the meniscal fragment being displaced and entrapped behind ACL.

X-ray (AP and lateral) views as shown in “Fig. 1” were completely normal. Analgesia, instruction of ice padding, and orthopedic follow-up were the initial management. **In clinic, detailed history was taking in which he reported improvement of his left knee pain and swelling. Unremarkable past medical and surgical history with no known allergies.** Physical examination of his left knee showed tenderness at the lateral joint line, limited knee extension (from 15° to 130°) compared to the right knee (from 0 to 130°). Thessaly [12] and McMurray tests were positive. Collateral and cruciate ligaments demonstrated no laxity with valgus stress, varus stress, anterior drawer, posterior drawer, Lachman and pivot-shift tests [[13], [14], [15]]. Plain radiographic images of his left knee were normal. MRI of left knee showed a long longitudinal tear of lateral meniscus extending from posterior horn to anterior horn. The medial meniscus, ligaments, and articular cartilage were normal. Coronal view of left knee showed entrapment of meniscal fragment in intercondylar notch represents “the fragment within the intercondylar notch sign” [7,16] and absence of lateral meniscal body in its anatomical position (Fig. 2A). On sagittal view, vertical tear in the peripheral zone of lateral meniscus (Fig. 2B). At the level of ACL on sagittal view, a meniscal fragment was seen posterior to ACL (Fig. 2C). These findings indicate displacement of lateral meniscal fragment toward intercondylar notch and entrapment behind and parallel to the ACL fibers which led to the appearance of double ACL. The patient consented for diagnostic arthroscopy of left knee which showed a bucket handle tear of lateral meniscus. The tear starts from the posterior horn of lateral meniscus extending to the beginning of anterior horn at the level of red-white zone (Fig. 3). The fragment was reduced to its anatomic position and repaired. **The procedure was done by the senior author with 15 years of arthroscopy experience** The patient was followed for two years. In last visit at our clinic, the patient was asymptomatic and able to achieve full range of motion with complete participation in sports activities.

## 3 Discussion

The advances in MRI granted us a sound knowledge about meniscal anatomy and pathology and facilitated correlation with arthroscopic findings [6,17]. Based on this, Multiple MRI signs of meniscal bucket-handle tears have been reported in the literature [5,18,19]. The double ACL sign can be caused by a bucket-handle tear of either medial meniscus with a meniscal fragment displaced and entrapped anterior to the ACL as reported by K. Takayama et al. [20] or by lateral meniscus [11]. In our case report, we present a bucket-handle tear of lateral meniscus with a displaced meniscal fragment entrapped posterior to the ACL.

Double posterior cruciate ligament sign “double PCL” was described by Singson et al. [21] and Weiss et al. [22] where the meniscal fragment is entrapped in front of PCL in the intercondylar notch and mainly involves medial meniscus. In our case, no meniscal fragment from lateral bucket-handle meniscal tear was displaced and entrapped in front of PCL as proven by MRI sagittal views and arthroscopic examination.

D.J. Dandy [23] examined arthroscopically 1000 symptomatic meniscal tears in his article and described that half width and half-length vertical tears of lateral menisci are the commonest types.

Terzidis et al. [24] evaluated 378 meniscal tears in stable knees and found 69.3% were located in the medial meniscus and 30.7% in the lateral meniscus. As well, he found the most frequent tear patterns of lateral menisci are radial (32.7%) and horizontal (25.8%).

Most reports describe a bucket-handle tear in medial meniscus with a displaced meniscal fragment entrapped in intercondylar notch in front of PCL [[25], [26], [27], [28]]. In our case, the unique and infrequent mechanism led to a bucket-handle tear involving lateral meniscus with a meniscal fragment entrapped in an unusual place intra-articularly behind ACL giving the appearance of a rare MRI sign “double ACL sign”.

In conclusion, detached ends of meniscal tears can assume various positions relative to cruciate ligaments. Double ACL sign on MRI images can be indicative of meniscal tear with marked displacement within the intercondylar notch.

## Ethical approval

We have reported a single case with no requirement for ethical approval. This manuscript does not describe a clinical study.

## Funding

No specific grant from funding agencies in the public, commercial, or not-for-profit sectors was received for this work.

## Author contribution

Adel Alahaidib-literature review and writing introduction.

Yasser alshehab – writing the case details.

Ahmed alajlan – following the patient.

Abdulaziz alahaideb-surgeon in charge.

Hamza Alrabia-editing and article reviewing.

All authors approved the final manuscript.

## Registration of research studies

We have reported a single case with no requirement for registry. This manuscript does not describe a clinical study.

## Guarantor

Adel Alahaidib (Sports Medicine fellow).

## Consent

Informed parental consent for the publication of this work was

Given on behalf of the patient.

## Provenance and peer review

Not commissioned, externally peer-reviewed.

## Declaration of competing interest

None.
